# Vick’s Illustrated Monthly Magazine

**Published:** 1878-03

**Authors:** 


### VICK'S ILLUSTRATED MONTHLY MAGAZINE.

We are pleased to welcome Vick's Illustrated *Monthly* Magazine. We presume it is to take the place of the Quar.

terly, that has proved so pleasant a visitor to us in days gone by, and we are glad to receive a monthly instead of a quarterly visit. The Magazine is only $1.25 per year, and we can assure any who take it, they will be amply repaid.

We have also received Vick's Catalogue and Floral Guide, in which can be found all classes of flowers, arranged systematically, and no one will find any trouble in selecting what they want.

We will say to our friends of the REGISTER, that nothing can be more appropriate to the dentist than the cultivation of flowers, even a few pots of flowers growing in a dental office will be appreciated by all.

